# The Role of Inflammatory and Hemostatic Markers in the Prediction of Severe Acute Pancreatitis: An Observational Cohort Study

**DOI:** 10.2174/0127722708356543241209060544

**Published:** 2024-12-17

**Authors:** Liudmila Orbelian, Nikita Trembach, Vladimir Durleshter

**Affiliations:** 1 Department of Surgery, Kuban State Medical University, Krasnodar Region, Russia;; 2 Department of Anesthesiology, Kuban State Medical University, Krasnodar Region, Russia

**Keywords:** Acute pancreatitis, hypercoagulation, thromboelastogram, pancreatitis, BISAP scale, SOFA scale

## Abstract

**Introduction:**

Acute pancreatitis (AP) is a serious inflammatory disease of the pancreas that can lead to significant morbidity and increased mortality. The special role of inflammation and disruption of the hemostatic system in the development of severe forms of the disease is known, however, the relationship between inflammatory and anti-inflammatory cytokines and thromboelastogram parameters has not been sufficiently studied.

**Aim:**

The aim of this study is to assess the prognostic significance of thromboelastogram parameters, interleukin-6, and interleukin-22 levels in assessing the risk for developing severe forms of acute pancreatitis.

**Materials and Methods:**

Data from 149 patients with acute pancreatitis were included in the analysis. The classification of AP was performed according to the 2012 Revision of the Atlanta Classification. Data including gender, age, lab tests, radiological information, and prognosis were included. The following scales were used to assess severity: SOFA scale and BISAP scale. IL-6 and IL-22 were analyzed at 24 h and 48 h after the onset of symptoms. The collected TEG parameters included K-time, R-value, and Maximum amplitude value at admission. All patients were divided into three groups: mild, moderate-severe, and severe pancreatitis.

**Results:**

Statistically significant differences were found between the groups in the IL-6 level at the first measurement and on day 2 of the study. IL-22 values were also higher in the group with severe pancreatitis, however, on day 2, its level became lower compared to the group of patients with moderate and mild pancreatitis. Statistically significant differences were found in the level of K-time, R-value, Maximum amplitude, fibrinogen concentration, and platelets count, demonstrating a hypercoagulation state in severe pancreatitis at admission. The conducted logistic regression showed that the factors associated with the development of severe forms are the number of points on the BISAP scale, the level of interleukin-6 in the first 24 hours of the disease, delta IL-22, and K-time. (AUC = 0.948).

**Conclusion:**

The study highlights that both IL-6 and IL-22 play crucial roles in the inflammatory cascade of severe acute pancreatitis. Their levels, along with specific hemostasis parameters like K-time and BISAP score, serve as reliable early predictors of disease severity.

## 1 INTRODUCTION

Acute pancreatitis (AP) is a serious inflammatory disease of the pancreas that can lead to significant morbidity and increased mortality. Timely and accurate assessment of the risk of developing severe forms of AP is critical for making adequate clinical decisions and optimizing treatment. In this regard, the study of prognostic markers that can predict the outcome of the disease is of significant clinical importance. Among the many biomarkers, thromboelastogram (TEG) and interleukin (IL) levels are of particular interest. TEG is a real-time hemostasis assessment method that allows the investigation of various aspects of the coagulation system, including reaction time, coagulation time, clot strength, and its degradation. Several studies have shown that disturbances in the hemostatic balance can be associated with the severity of AP and its complications [1, 2]. In particular, hypercoagulation revealed by TEG can serve as a marker of severe disease and the development of necrotic pancreatitis [1, 3, 4]. In addition, interleukins, as key mediators of the inflammatory response, play an important role in the pathogenesis of AP [5]. Increased levels of proinflammatory interleukins, such as IL-6, IL-8, and IL-1β, correlate with the severity of the inflammatory process and the development of systemic complications [6, 7]. Interleukin-6 (IL-6) stands out among others since it is involved in the activation of C-reactive protein and other markers of systemic inflammation, which are also associated with the prognosis of the disease [8, 9]. IL-6 is a well-established pro-inflammatory cytokine that is rapidly produced in response to tissue injury and infection. Elevated levels of IL-6 have been correlated with the severity of inflammation and are considered a marker for systemic inflammatory response syndrome (SIRS), which is often associated with severe pancreatitis.

IL-22, a member of the IL-10 cytokine family, has a dual role in inflammation and tissue regeneration. It is produced by immune cells and acts primarily on epithelial cells, promoting antimicrobial defense, cell survival, and tissue repair [10]. The involvement of IL-22 in pancreatitis is less studied, but emerging evidence suggests it may have a protective role in pancreatic tissue during inflammation [11]. Furthermore, by investigating IL-6 and IL-22 across mild, moderate-severe, and severe cases of pancreatitis, we aimed to assess whether levels of these cytokines correlate with the clinical severity of pancreatitis, which could enhance understanding of the disease's pathophysiology and explore the potential of IL-6 and IL-22 as independent biomarkers for early prediction of disease progression, which is crucial for timely and appropriate therapeutic interventions.

Additionally, due to the close relationship between systemic inflammation and the blood coagulation system, a combined study of TEG parameters and interleukin levels can provide a more complete picture of the pathophysiological processes occurring in AP and help in predicting its outcome. At the same time, the integration of these markers into clinical practice requires a thorough analysis of their prognostic significance.

The prognostic significance of TEG and interleukin levels has been previously studied in the context of various inflammatory diseases and critical conditions such as sepsis and trauma [12, 13]. However, there is a lack of such studies in AP. Our study aims to fill this gap and provide new data on the relationship between these markers and the severity of pancreatitis.

The goal of this study is to evaluate the prognostic significance of inflammatory markers (interleukin-6 and interleukin-22), their dynamics and hemostatic parameters in predicting the development of severe acute pancreatitis (SAP). Additionally, by integrating these biomarkers with clinical scoring systems like the BISAP score, the study aims to develop a comprehensive predictive model that enhances early risk stratification, facilitates timely therapeutic interventions, and improves outcomes in patients with acute pancreatitis.

## 2 MATERIALS AND METHODS OF THE STUDY

The prospective observational cohort study was conducted at the clinic of the Kuban State Medical University in the Regional Clinical Hospital No. 2 of the city of Krasnodar in patients admitted with acute pancreatitis from 10 0f January 2024 to 31 July 2024.

### 2.1 Inclusion Criteria and Patient’s Enrollment

Patients admitted with a diagnosis of acute pancreatitis were consecutively assessed for inclusion in the study. The diagnostic criteria of AP included two out of the following three characteristics: (1) abdominal pain; (2) serum amylase and/or lipase ≥3 times the upper limit of the normal value; and (3) characteristic pancreatic signs revealed by ultrasound, computer tomography or magnetic resonance imaging. The classification of AP was performed according to the 2012 Revision of the Atlanta Classification. All patients were divided into three groups: mild (MAP), moderate-severe (MSAP), and severe pancreatitis (SAP).

Exclusion criteria included (1) patients younger than 18 years old or older than 80 years old; (2) patients admitted beyond 24 hours after symptom onset; (3) pregnant patients; and (4) patients with malignant diseases, autoimmune diseases, or other severe comorbidities, loss of data, violation of the technique for storing biomaterial and laboratory samples, chronic pancreatitis. Individuals with incomplete follow-up were excluded.

Data including gender, age, lab tests, radiological information, and prognosis were included. This study was approved by the Institutional Review Board of Kuban State Medical University (Statement No. 113 dated 12.09.2022).

The work was funded by the Russian Science Foundation (Research Project No. 24-25-00164).

### 2.2 Clinical and Laboratory Assessment

The following scales were used for initial assessment: SOFA scale and BISAP scale, from laboratory markers, the level of leukocytes, C-reactive protein, lactate, and procalcitonin were assessed.

Blood Samples and Biomarkers Plasma samples were collected upon admission and daily for the following 2 days. The exact time for each blood sampling was registered. The blood samples were collected in plasma separator tubes (containing Lithium-Heparin gel), centrifuged (2000 rounds, 25°C, 10 min), and stored at -80°C until analyzed. IL-6 and IL-22 were analyzed using a human proinflammatory ultrasensitive kit (SEC032Hu ELISA Kit for Interleukin 22 (IL22) and SEA079Hu ELISA Kit for Interleukin 6 (IL6), CLOUD-CLONE CORP (CCC, USA)). The analyses were assessed according to the manufacturer’s instructions. Admission data (24h) and data on a second day (48h) after the onset of the disease were collected. Procalcitonin and C-reactive peptide (CRP) were analyzed in accordance with the certified standard analysis in a hospital.

Venous blood (6 ml) before initiation of treatment was collected for the TEG test. The collected TEG parameters included K-time, R-value, and Maximum amplitude (MA) value (TEG 5000, Haemoscope (USA)). The fully automatic blood coagulation analyzer was used to measure activated partial thromboplastin time (APTT), prothrombin time (PT), D-dimer and fibrinogen (FIB) concentration, and platelet count.

The work was carried out in accordance with the STROBE guidelines for the publication of observational studies.

### 2.3 Statistical Analysis

Taking into account the fact that it was planned to study 2 interleukins and the prevalence of severe forms of 20%, the planned sample was 100 patients. Taking into account the variability in the number of predictors and the frequency of severe forms, the total target sample size was 150 patients. Statistical Analysis of the data distribution was assessed using the Kolmogorov-Smirnov test. Differences in biomarker levels between paired samples taken at 0-24 h and 25-48 h were analyzed using the Wilcoxons test. Вiomarkers were analyzed at 0-24 h (IL-6 24 h and IL-22-24 h) and 25-48 h (IL-6 48 h and IL-22 48 h) for each severity category. The difference between the mean values of 0-24 h and 25-48 h was denominated the delta-value. Also, for each severity group of patients, the degree of change in the level of interleukins was calculated in relation to the value obtained in the first measurement (delta IL-6/IL-6 24 h and delta IL-22/IL-22). Differences between interleukin levels, delta-values, and differences between the degree of each severity group were analyzed using the Kruskal-Wallis test. Post-hoc analysis was carried out using the Dunn test; a significance level of less than 0.05 was considered statistically significant. Chi-square tests was used to compare categorical variables. ROC curves (with severe AP as outcome) were performed to calculate an area under curve (AUC) and cut-off levels for each value. For further analysis of the association between markers and disease severity, univariate and multivariate (adjusted for age and gender) logistic regression analyses were performed. All statistical analysis was executed using MedCalc v22.020 (MedCalc Software Ltd., Belgium).

### 2.4 Source Bias

#### 2.4.1 The Following Measures have been Taken to Limit Source Bias

Selection Bias: This was managed by clearly defining inclusion and exclusion criteria that were strictly followed. The study included consecutive patients presenting with AP to reduce selection bias.

Measurement Bias: In addition, to reduce measurement bias, standardized protocols for the measurement of biomarkers and hemostasis parameters were used. All laboratory tests were performed in a single, centralized lab to reduce variability in test results. The equipment was calibrated, and the personnel involved in biomarker measurements were trained.

Confounding Variables: Multivariate analysis was performed to adjust for potential confounding factors.

Statistical Analysis: Appropriate statistical tests were chosen based on the distribution of data and the study design.

## 3 RESULTS OF THE STUDY

**Fig. (1):**
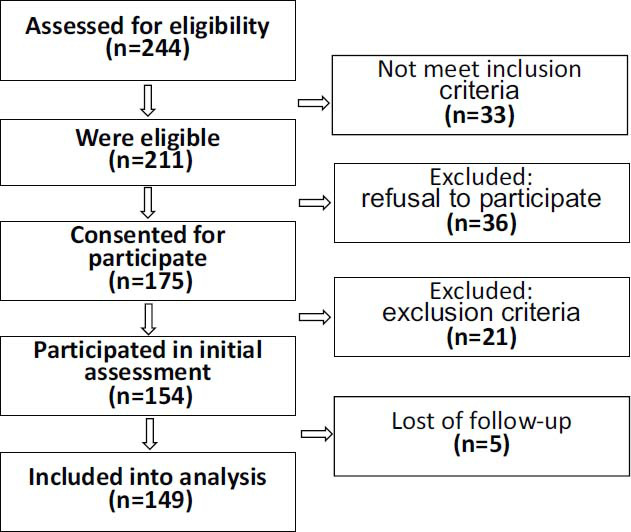
Flowchart of patient selection and inclusion in the study.

The main characteristics of the studied group of patients are presented in Table **1**. In total, data from 149 patients were included in the analysis (Fig. **1**).

This flowchart illustrates the patient recruitment and selection process for the study evaluating inflammatory and hemostatic markers in acute pancreatitis (AP). Initially, all patients admitted with a diagnosis of AP were assessed for eligibility (n=244). Patients were included if they had a confirmed diagnosis of acute pancreatitis based on abdominal pain, serum amylase or lipase levels ≥3 times the upper limit of normal, and/or imaging findings consistent with pancreatitis, with classification according to the 2012 Revised Atlanta Classification. A total of 211 patients met the inclusion criteria. Thirty-six patients refused to participate, 21 patients were excluded due to addition more than 24 hours after symptom onset (n=16), pregnancy (n=1), malignant (n=3) or autoimmune (n=1) diseases, and 5 patients were excluded due to or follow-up loss.

Severe pancreatitis was recorded in 23 patients (15.5%), moderate pancreatitis - in 41 patients (27.5%), and mild pancreatitis was recorded in 85 cases (57%). Mortality was 8% (12 deaths, all in the severe pancreatitis group, which ultimately amounted to 52% in this group). There were no statistically significant differences in etiology between the subgroups; in all groups, as well as in general, biliary etiology of the disease prevailed. The studied subgroups also did not differ in age, as well as in the level of laboratory markers, such as procalcitonin, CRP, and leukocytes. Statistically significant differences were found in the SOFA and BISAP scores; in patients with mild pancreatitis, the scores on these scales upon admission were lower compared to other groups; however, there were no differences between patients in the moderate and severe groups.

Statistically significant differences were found between the groups in the IL-6 level at the first measurement and on day 2 of the study, but the groups did not differ in the delta level and the increase in the cytokine level. IL-22 values were also higher in the group with severe pancreatitis, however, on day 2, its level became lower than in the group of patients with moderate and mild pancreatitis. Moreover, although the concentration of IL-22 decreased in all patients, the delta and the degree of decrease were significantly higher in severe pancreatitis (Table **2**).

**Fig. (2):**
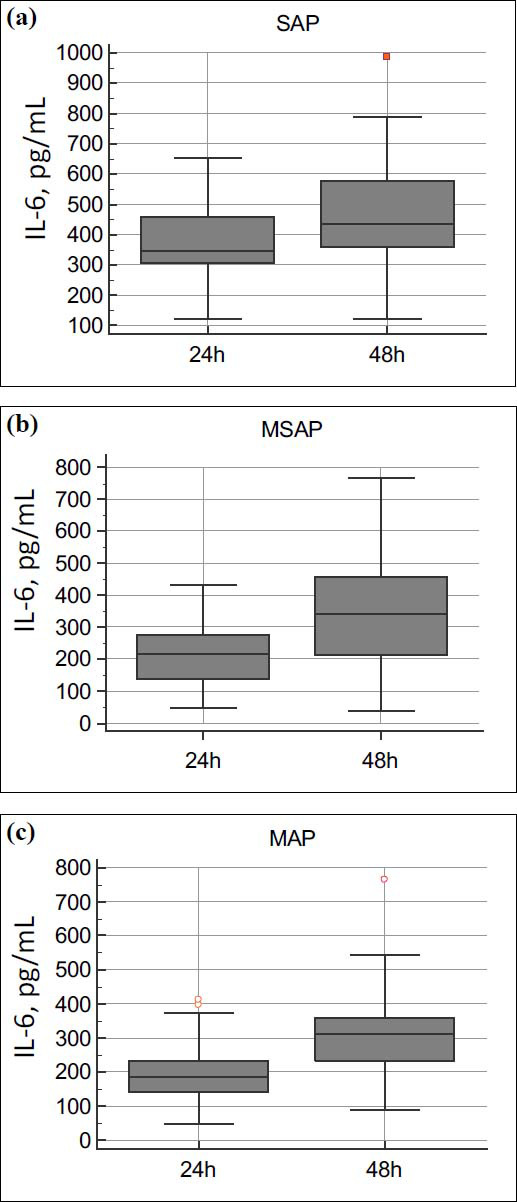
(**a-c**). Dynamics of interleukin-6 (IL-6) levels across different severity groups of acute pancreatitis.

In all subgroups, a statistically significant increase in IL-6 levels was noted (*p* =0.0004, *p* =0.0003 and *p* <0.0001 for severe, moderate and mild pancreatitis) (Fig. **2**).

This figure demonstrates the changes in interleukin-6 (IL-6) levels over 48 hours in patients with acute pancreatitis (AP) divided into three severity groups: mild acute pancreatitis (MAP, n=85), moderate-severe acute pancreatitis (MSAP, n=41), and severe acute pancreatitis (SAP, n=23).

• **Panel 2a:** In SAP patients (n=23), IL-6 levels significantly increased from a median of 347 pg/mL at 24 hours to 434 pg/mL at 48 hours (*p*< 0.0001). These levels were significantly higher than in both the MSAP and MAP groups at all time points (*p*< 0.0001).

• **Panel 2b:** In MSAP patients (n=41), IL-6 levels rose from 214 pg/mL at 24 hours to 342 pg/mL at 48 hours (*p*= 0.0003). These levels were intermediate between MAP and SAP groups and significantly higher than MAP at both time points (*p*< 0.05).

• **Panel 2c:** In MAP patients (n=85), IL-6 levels increased from 185 pg/mL at 24 hours to 312 pg/mL at 48 hours (*p*= 0.0004). IL-6 levels in this group remained significantly lower than in both SAP (*p*< 0.0001) and MSAP (*p*< 0.05) groups.

Differences between groups were evaluated using the Kruskal-Wallis test (*p*< 0.05), followed by Dunn's post-hoc test for intergroup comparisons. Data are presented as medians and interquartile ranges to account for non-parametric distributions.

This figure underscores IL-6 as a reliable biomarker for distinguishing the severity of AP. Patients with SAP exhibit consistently elevated IL-6 levels, correlating with systemic inflammation and disease progression. Monitoring IL-6 dynamics provides a valuable tool for early risk stratification and targeted clinical management in AP.

**Fig. (3):**
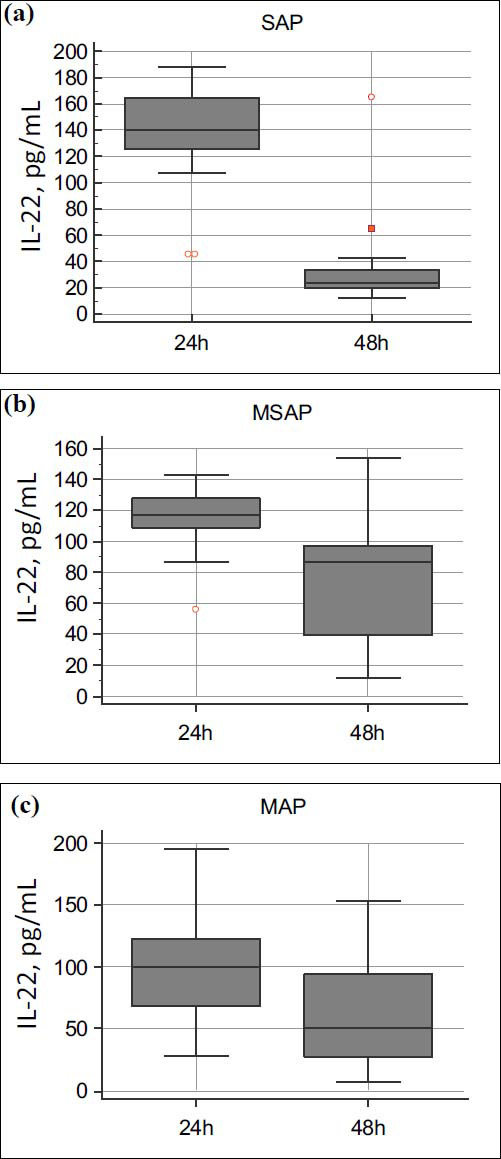
(**a-c**). Dynamics of interleukin-22 (IL-22) levels across different severity groups of acute pancreatitis.

The opposite dynamics were noted in the level of interleukin-22 - all subgroups showed a statistically significant decrease in its value on the second day of observation (*p* <0.0001 for all severity subgroups) (Fig. **3**).

This figure illustrates the changes in interleukin-22 (IL-22) levels over 48 hours in patients with acute pancreatitis (AP), categorized into three severity groups: mild acute pancreatitis (MAP, n=85), moderate-severe acute pancreatitis (MSAP, n=41), and severe acute pancreatitis (SAP, n=23).

• **Panel 3a:** In SAP patients (n=23), IL-22 levels significantly decreased from a median of 140 pg/mL at 24 hours to 23 pg/mL at 48 hours (*p*< 0.0001). The reduction (ΔIL-22) was notably greater compared to the MSAP and MAP groups (*p*< 0.0001).

• **Panel 3b:** In MSAP patients (n=41), IL-22 levels decreased from 117 pg/mL at 24 hours to 87 pg/mL at 48 hours (*p*< 0.0001). This decline was less pronounced than in the SAP group but greater than in the MAP group (*p*< 0.05).

• **Panel 3c:** In MAP patients (n=85), IL-22 levels decreased from 100 pg/mL at 24 hours to 50 pg/mL at 48 hours (*p*< 0.0001). The reduction was smaller than in both the SAP and MSAP groups (*p*< 0.05).

Statistical differences between groups were assessed using the Kruskal-Wallis test (*p*< 0.05), followed by Dunn’s post-hoc test for pairwise comparisons. Data are presented as medians with interquartile ranges to reflect non-parametric distributions.

This figure highlights the predictive and potentially protective role of IL-22 in acute pancreatitis. Patients with SAP exhibit the highest initial IL-22 levels at 24 hours, followed by a sharp decline by 48 hours. The degree of reduction (ΔIL-22) correlates with disease severity, suggesting that IL-22 dynamics could serve as a prognostic biomarker for severe AP. These findings may also point to IL-22’s role in the balance between inflammation and tissue repair during the acute phase of pancreatitis.

When analyzing the parameters of the hemostasis system, statistically significant differences were found in the level of K-time, R-value, Maximum amplitude, fibrinogen concentration, and platelets count, demonstrating a hypercoagulation state in severe pancreatitis at admission (Table **3**).

Based on the ROC analysis of the prognostic value of the parameters that showed differences in the subgroups, we identified several factors that showed good prognostic value. IL-6 and IL-22 at admission, delta IL-22, delta IL-22/IL-22 24h, K-time and BISAP score showed an AUROC more than 0,8 (Table **4**).

Figs. (**4** and **5**) show a graphical representation of the rock curves of the specified parameters.

This figure presents the ROC curves for interleukin-6 at 24 hours (4a), interleukin-6 at 48 hours (4b), interleukin-22 at 24 hours (4c), interleukin-22 at 48 hours (4d), their delta changes (ΔIL-6 (4e) and ΔIL-22 (4f)), Delta IL-6/IL-6 at 24 hours ratio (4g) and Delta IL-22/IL-22 at 24 hours ratio (4h) in predicting severe acute pancreatitis (SAP). The analysis demonstrates the predictive accuracy of these biomarkers measured within the first 48 hours after symptom onset in differentiating SAP from milder forms of the disease.

IL-6 at 24 hours and IL-22 at 24 hours are both strong predictors of SAP, with high AUC values and sensitivity. The delta change in IL-22 (ΔIL-22) provides additional prognostic accuracy, highlighting its potential role in identifying patients at risk of severe disease progression.

This figure underscores the importance of early measurement of IL-6 and IL-22, as well as monitoring their dynamic changes, for predicting SAP. The strong performance of ΔIL-22 as a predictive marker suggests that not only absolute cytokine levels but also their temporal trends are critical in identifying high-risk patients, enabling timely and targeted interventions.

This figure presents ROC curves for α-Angle (5a), Fibrinogen Level (5b), K-Time (5c), Maximum Amplitude (MA) (5d), Platelet Count (5e), R-Time (5f), BISAP Score (Bedside Index for Severity in Acute Pancreatitis) (5g) SOFA Score (Sequential Organ Failure Assessment) (5hin predicting the development of severe acute pancreatitis (SAP). These metrics were evaluated for their predictive accuracy using the area under the curve (AUC). This figure highlights the complementary value of hemostasis parameters and clinical scoring systems in predicting SAP. While TEG parameters such as K-time and MA exhibit strong predictive performance, the BISAP score remains a practical and efficient tool for early risk assessment   in   clinical   settings. Platelet count, although useful as part of a broader panel, shows limited standalone predictive accuracy. Integrating these parameters can enhance the early identification of SAP, enabling timely interventions to improve patient outcomes.

The conducted logistic regression showed that the factors associated with the development of severe forms are the number of points on the BISAP scale, the level of interleukin-6 in the first 24 hours of the disease, delta IL-22, and K-time. Variables not included in the model were α-angle, fibrinogen level, IL-22 level in admission, IL-6 level on day 2, maximum amplitude, R-value and SOFA score (Table **5** and **6**).

**Fig. (6):**
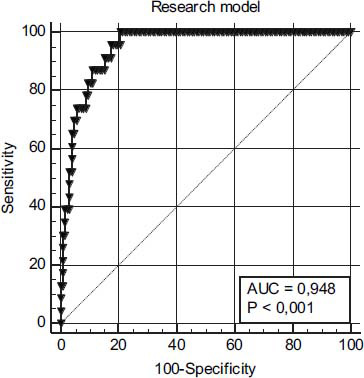
Receiver operating characteristic (ROC) curve for the combined predictive model in severe acute pancreatitis (SAP).

Hosmer-Lemeshow test showed an excellent good-ness-of-fit (Chi-squared 1.5886, DF=8, *p* = 0.9911). ROC curve analysis demonstrated good predictive ability of the obtained model Area under the ROC curve (AUC) = 0.948 ± 0.017 (Fig. **6**), cut-off point >-1.618 (16.5%) (sensitivity 100%, specificity 80%).

This figure illustrates the ROC curve for the combined predictive model, which integrates multiple significant biomarkers and clinical parameters, to assess its diagnostic performance in predicting severe acute pancreatitis (SAP). The model includes the BISAP score, interleukin-6 (IL-6) levels at 24 hours, the delta change in interleukin-22 (ΔIL-22), and the thromboelastography (TEG) parameter K-time. The ROC curve demonstrates that the combined model outperforms individual parameters in predicting SAP severity. Moreover, by integrating inflammatory, hemostatic, and clinical data, the model provides a robust and reliable tool for early risk stratification.

The high AUC value and exceptional sensitivity of this combined predictive model highlight its potential for clinical use in the early identification of patients at risk of SAP. Early detection enables prompt and targeted interventions, reducing the risk of complications and mortality associated with SAP. This integrative approach underscores the value of combining biomarkers with clinical scoring systems to improve prognostic accuracy in acute pancreatitis.

## 4 DISCUSSION

Based on the results, the study underscores the significant role of interleukins, particularly IL-6 and IL-22, as well as specific hemostasis parameters, in the development and prognosis of severe acute pancreatitis (SAP).

### 4.1 Role of Cytokines in Severity of Acute Pancreatitis

IL-6 levels were significantly elevated in patients with severe acute pancreatitis compared to those with milder forms. This elevation was noted both in the initial 24 hours and later at 48 hours. Higher IL-6 concentrations were strongly associated with the severity of the disease (*p* <0.0001). The ROC curve analysis demonstrated a good predictive capacity for IL-6 in identifying SAP, with high sensitivity (87%) and specificity (80%) at 24 hours.

Interleukin-6 (IL-6) plays a complex role in acute pancreatitis. Contrary to expectations, IL-6 deficient mice exhibited more severe tissue injury and higher mortality rates in cerulein-induced pancreatitis, suggesting an anti-inflammatory role for endogenous IL-6 [14]. However, IL-6 is also implicated in disease aggravation through JAK/STAT pathway activation [15]. In clinical studies, serum IL-6 levels are significantly better than traditional inflammatory markers routinely used in the clinic, such as C-reactive protein (CRP), and appear to be the most promising as an early predictor of the severity of acute pancreatitis [16] with an AUC 0.8 for the development of a severe form [17]. IL-6 concentrations peaked earlier than CRP, offering comparable but earlier severity prediction [18]. These findings highlight the complex and multifaceted role of IL-6 in acute pancreatitis pathogenesis and its potential as a biomarker for disease severity. Our work confirms the important role of IL-6 in acute pancreatitis and its prognostic significance. In the work of Kobler *et al*., an increase in IL-6 levels was noted starting from the first day of the disease, while the initial IL-6 level was statistically significantly higher in severe pancreatitis [8], which was similar to our study. Nevertheless, the authors showed that the difference between the value on the second day compared with the first also differed in subgroups of patients with different severity. We did not find such a pattern – no differences were found in absolute values; however, we found a statistically significant difference in the conditional value – the ratio of the delta of the IL to the initial value.

IL-22 showed a different trend - its concentration was statistically higher in patients with severe forms of the disease, with a notable decrease in its levels after 48 hours across all subgroups. IL-22 also had strong predictive value, particularly for the first 24 hours, with 87% sensitivity and 75% specificity. Interestingly, a significant drop in IL-22 levels between the 24-hour and 48-hour periods was a predictive factor for SAP. Our data showed that in the initial stages, the level of IL-22 is also quite high and higher in patients with severe forms of the disease. Jin *et al*. demonstrated a similar dynamic of IL-22 levels – an initial increase reflecting a compensatory reaction of the anti-inflammatory system followed by a fairly rapid decrease [19]. Our data confirm this pattern, while the level of interleukin-6 remained at the same level, and the degree of decrease in IL-22 was associated with the severity of the course – the more pronounced the decrease was observed in patients with the more severe course of pancreatitis. The patterns we have identified indicate that the severity of the course may probably depend on the possibility of activating the anti-inflammatory system and its ability to maintain this activation in the acute period.

Interleukin-22 (IL-22) plays a dual role in acute pancreatitis, acting as a marker of disease severity and as a protective agent. IL-22 levels in the blood of patients with pancreatitis correlate with the severity of the disease and the degree of organ dysfunction, making it an important prognostic marker [19]. In addition, IL-22 protects pancreatic acinar cells from damage by suppressing inflammation and regulating autophagy and promotes intestinal barrier restoration [20]. This confirms its important role in protecting organs from inflammation-induced damage [21]. Our results confirm a potential protective role of IL-22 in acute pancreatitis, which makes it not only a prognostic marker but also a promising therapeutic agent, which requires further study [19, 22].

### 4.2 Role of Coagulation in the Severity of Acute Pancreatitis

The analysis of hemostasis parameters such as K-time, MA, and platelet counts also revealed notable differences between patients with mild and severe pancreatitis. A shortened K-time and elevated MA were characteristic of patients with SAP. These factors were statistically significant (*p* <0.0001) and demonstrated good predictive capacity in distinguishing the severity of pancreatitis. The fibrinogen levels were significantly higher in SAP patients, indicating a hypercoagulable state, which is typical of severe systemic inflammation. Thromboelastography (TEG) has emerged as a valuable tool for evaluating coagulation disorders in acute pancreatitis (AP). Studies have shown that TEG parameters, including clot reaction time (R-value), clot generation time (K-value), maximum amplitude, and α-angle, significantly differ between AP severity groups and correlate with disease severity [23, 24]. AP induces a transient hypercoagulable state characterized by increased MA and coagulation index, elevated platelet aggregation, and higher circulating tissue factor levels [25]. This hypercoagulability is supported by earlier findings of elevated coagulation indices, hyperfibrinogenemia, and accelerated fibrinogen conversion rates in AP patients [26]. Combining TEG with routine coagulation tests provides a comprehensive assessment of coagulation profiles in AP, potentially aiding in early diagnosis and prognosis evaluation [24]. These findings underscore the importance of monitoring coagulation parameters in AP management. In pancreatitis, inflammation, and activation of coagulation are interrelated and enhance pathological processes. Inflammation causes vascular endothelial damage and activation of thrombus formation *via* tissue factor pathway and thrombin, which in turn enhances inflammation. Thrombin activates PAR receptors, causing platelet aggregation and release of proinflammatory cytokines such as IL-6. In severe cases of pancreatitis, the systemic inflammatory response and activation of coagulation can lead to thrombosis, which aggravates microcirculatory disorders and organ damage [2]. Therefore, anticoagulants such as low molecular weight heparin can potentially reduce the severity of inflammation and improve the prognosis of patients with pancreatitis [2]. However, the question of specific hemostatic system indices that could be relied upon when considering the use of drugs that reduce coagulation activity remains open. Apparently, routine markers such as APTT and PT have shown themselves to be poor prognostic factors [2]. Despite the fact that many parameters differed in patients with different severity of pancreatitis, only the K-time value was identified as an independent predictor of the development of a severe form of pancreatitis. Similar results were obtained in the work of C. Fan *et al*., who used rapid thromboelastography and identified K-time as an early predictor of pancreatitis severity along with neutrophils, triglycerides, and amylase [24]. Certainly, the early stage is characterized by hypercoagulation, and with the progression of inflammation and thrombosis, hemostasis patterns characteristic of depletion of coagulation factors may be observed, and signs of disseminated intravascular coagulation syndrome may appear [25, 27]. In this case, D-dimer may be probably a good prognostic marker in acute pancreatitis [28]. However, this occurs in the later stages of the disease. Thus, the early stage of pancreatitis is characterized by an increase in the coagulation status without pronounced thrombus formation, which is a good opportunity to identify these disorders using TEG and prevent subsequent thrombus formation leading to organ dysfunction.

The combined analysis of interleukins (particularly IL-6 and IL-22), hemostasis parameters (K-time), and clinical scales such as BISAP showed excellent predictive power for SAP, with the model's AUC reaching 0.948. The model's sensitivity and specificity suggest that it could be highly effective for early prediction and stratification of patients with severe pancreatitis, allowing for timely and targeted interventions.

## 5 LIMITATIONS

The study was single-center, and population characteristics may contribute to the course of the disease, as well as the structure of the etiology of pancreatitis. From the entire spectrum of proinflammatory markers, we selected interleukin-6 as the most frequently mentioned and proven early marker, and other cytokines, such as interleukin-1 and TNF, were not assessed. The dynamics of interleukins and hemostasis indicators after 48 hours of illness were not studied, which may also be of interest and is the subject of further study.

## CONCLUSION

The study highlights that both IL-6 and IL-22 play crucial roles in the inflammatory cascade of severe acute pancreatitis. Their levels, along with specific hemostasis parameters like K-time and BISAP score, serve as reliable early predictors of disease severity. These findings suggest that monitoring of these biomarkers in clinical practice could improve the early detection and management of SAP, reducing the risk of mortality and complications associated with the disease. It should be noted that a decrease in the concentration of IL-22 is associated with a greater risk of developing a severe form of the disease, which may indicate its important protective role and a potential therapeutic agent. We hope that the results of our study can contribute to improving prognostic models for AP and support the development of more effective treatment and monitoring strategies for patients with this disease.

## AUTHORS’ CONTRIBUTIONS

Idea/Concept: VD, LO; Design: LO; Control/Supervision: VD; Literature Review: LO, NT; Writing The Article: LO, NT; Critical Review: VD; LO is the main author in this study, and all authors have read and approved the final manuscript.

## Figures and Tables

**Fig. (4):**
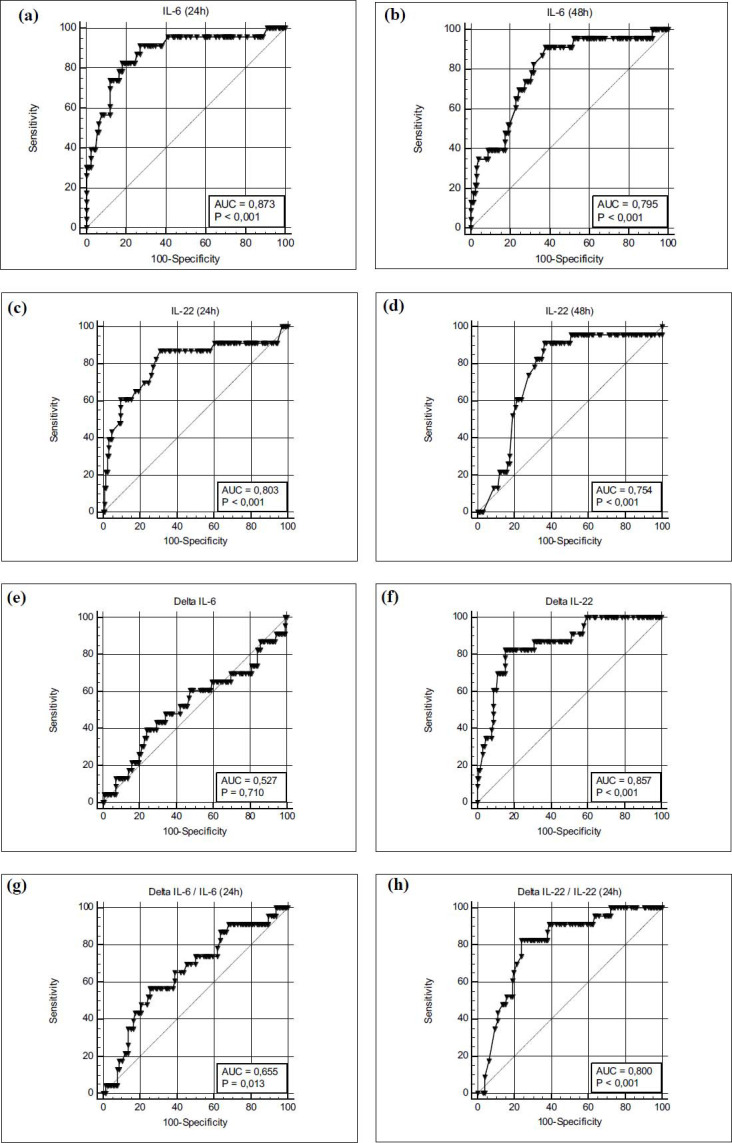
**(a-h)** Receiver operating characteristic (ROC) curves for interleukin levels and their dynamics in predicting severe acute pancreatitis (SAP).

**Fig. (5):**
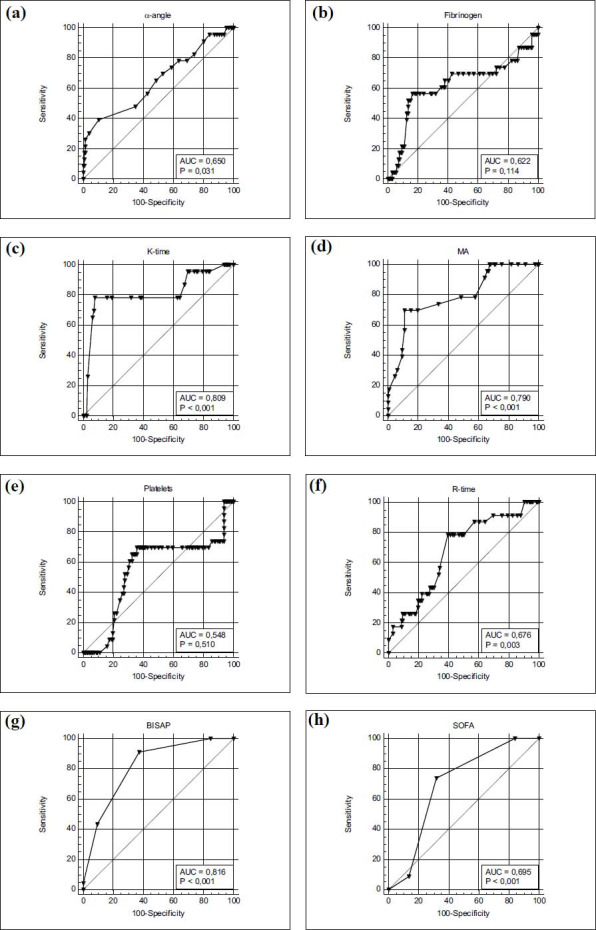
**(a-h)** Receiver operating characteristic (ROC) curves for hemostasis parameters and clinical scoring systems in predicting severe acute pancreatitis (SAP).

**Table 1:** Characteristics of patients.

**-**	**MAP (n=85)**	**MSAP (n=41)**	**SAP (n=23)**	***p***
Etiology	-	-	-	-
Biliary, (n)	10	18	38	0.99
Alcoholic, (n)	8	10	25	0.67
Other, (n)	5	13	23	0.68
Gender, male (n)	48	22	10	0.54
Age, year	46 (43-50)	48 (41-54)	47 (38-57)	0.71
SOFA, score	1 (1-1)	2 (2-3)*	2 (2-3)*	*p* <0.0001
BISAP, score	1 (1-1)	2 (1-2.25)*	2 (2-3)*	*p* <0.0001
C-reactive peptide, mg/L	80 (70-94)	88 (75-94)	100 (63-113)	0.1
Procalcitonin, μg/L	1.2 (0.8-1.5)	1.1 (1.0-1.9)	1.2 (1-1.9)	0.45
Hours from onset to admission	14 (6-21)	15 (6-22)	14 (5-21)	0.43
Mortality	0	0	12	*p* <0.0001

Note: * - *p* <0.05 compared to MAP.

**Table 2:** Comparison of interleukin levels in patients with different courses of acute pancreatitis.

**Parameter**	**MAP**	**MSAP**	**SAP**	***p***
IL-6 (24 h), pg/mL	185 (141-235)#	214 (137-275)#	347 (306-455)	*p *<0.0001
IL-6 (48 h), pg/mL	312 (233-359)#	342 (211-456)#	434 (361-575)	*p *<0.0001
Delta IL-6, pg/mL	108 (43-211)	114 (13-250)	108 (47-225)	0.91
Delta IL-6/IL-6 (24 h)	0.53 (0.34-0.80)	0.55 (0.03-1.18)	0.27 (0.11-0.63)	0.05
IL-22 (24 h), pg/mL	100 (68-123)#$	117 (109-128)#*	140 (125-164)*$	*p *<0.0001
IL-22 (48 h), pg/mL	50 (27-94)#	87 (39-97)#	23 (19-33)	*p *=0.0001
Delta IL-22, pg/mL	-34 (-74-(-2))#	-30 (-54-(-16))#	-108 (-140-(-94))	*p *<0.0001
Delta IL-22/IL-22 (24 h)	0.39 (0.02-0.71)#	0.28 (0.12-0.49)#	0.80 (0.69-0.88)	*p *<0.0001

**Note:** # - *p* <0.05 compare to SAP; $ - *p* <0.05 compare to MSAP; * - *p* <0.05 compared to MAP.

**Table 3:** Parameters of the hemostasis system in the studied groups.

**Parameter**	**MAP**	**MSAP**	**SAP**	***p***
R, min	7.7 (7.2-9.8)	9.8 (8.7-13.0)*	9.3 (9.0-11.2)*	*p *<0.0001
K-time, min	1.8 (1.5-1.8)#	2.0 (1.3-2.7)#	1.0 (0.9-1.2)	*p *<0.0001
α-angle, °	68 (67-75)	71 (69-74)	68 (64-71)	0.08
Maximum amplitude, mm	67 (65-68)#	58 (55-68)#	78 (73-80)	<0.0001
Lys30, %	3.3 (2.4-4.2)	3.4 (3.2-4.7)	3.7 (3.2-4.0)	0.57
Fibrinogen, g/L	5.0 (4.3-5.6)	6.0 (4.8-7.5)*	6.4 (4.0-7.0)*	0.0001
Platelets, ×10^9^/L	221 (191-233)	165 (123-217)	135 (124-249)	0.003
D-dimer, μg/mL	3.6 (3.1-4.2)	3.4 (2.9-4.0)	3.8 (3.3-4.2)	0.21
APTT, min	38.3 (32.5-44.3)	39.4 (33.8-43.1)	41.6 (33.1-45.3)	0.42
PTT, min	12.1 (11.3-13.7)	12.3 (11.7-13.9)	12.4 (11.6-13.8)	0.65

**Abbreviations:** APTT- Activated Partial Thromboplastin Time, PTT- Prothrombin Time; **Note:** # - *p* <0.05 compared to SAP; * - *p* <0.05 compared to MAP.

**Table 4:** Prognostic value of parameters in predicting severe acute pancreatitis.

**Parameter**	**AUC**	**SD**	***p***	**Cut-off point**	**Sensitivity, %**	**Specificity, %**
IL-6 (24 h), pg/mL	0.873	0.04	<0.0001	>279	83	82
IL-6 (48 h), pg/mL	0.795	0.05	<0.0001	>341	91	62
IL-22 (24 h), pg/mL	0.803	0.06	<0.0001	>122	87	75
IL-22 (48 h), pg/mL	0.754	0.05	<0.0001	≤43	91	64
Delta IL-6, pg/mL	0.527	0.07	0.71	≤52	39	76
Delta IL-22, pg/mL	0.857	0.04	<0.0001	≤-92	83	85
Delta IL-6/IL-6 24 h	0.655	0.05	0.013	≤0.31	57	75
Delta IL-22/IL-22 24 h	0.800	0.04	<0.0001	>0.65	83	76
α-angle, °	0.650	0.07	0.03	≤66	39	90
Fibrinogen, g/L	0.622	0.08	0.114	>6.3	56	83
K-time, min	0.809	0.06	<0.0001	≤1.2	78	92
Maximum amplitude, mm	0.790	0.05	<0.0001	>70	70	89
Platelets, ×10^9^/L	0.548	0.07	0.51	≤198	70	64
R, min	0.676	0.06	0.003	>8.9	78	60
BISAP, score	0.816	0.04	<0.0001	>1	91	62
SOFA, score	0.695	0.05	<0.0001	>1	74	68

**Table 5:** Logistic regression equation.

**Variable**	**Coefficient**	**Std. Error**	**Wald**	***p***
BISAP	2.73091	0.96	8.004	0.0047
IL-6 (24 h)	0.021563	0.007	7.8930	0.0050
Delta IL-22	-0.037377	0.014606	6.5490	0.0105
K-time	-2.93711	1.00369	8.5633	0.0034
Constant	-11.64892	3.98850	8.5301	0.0035

**Table 6:** Table odds ratios and 95% confidence intervals for predictors.

**Variable**	**Odds Ratio**	**95% CI**
BISAP, score	15.3	2.3 to 101.8
IL-6 (24 h), pg/mL	1.02	1.01 to 1.03
Delta IL-22, pg/mL	0.96	0.93 to 0.99
K-time, min	0.05	0.001 to 0.380

## Data Availability

The data used to support the findings of this study are available from the corresponding author upon request.

## LIST OF ABBREVIATIONS

AP: Acute Pancreatitis
APTT: Activated Partial Thromboplastin Time
AUC: Area Under Curve
BISAP: Bedside Index for Severity in Acute Pancreatitis
CRP: C-reactive Peptide
IL: Interleukin
MAP: Mild Acute Pancreatitis
MSAP: Moderate-severe Acute Pancreatitis
PTT: Prothrombin Time
ROC: Receiver Operating Characteristic Curve
SAP: Severe Acute Pancreatitis
SOFA: Sequential Organ Failure Assessment Score
TEG: Thromboelastography

## STANDARDS OF REPORTING

STROBE guidelines were followed.
